# Diabetic Macular Edema Optical Coherence Tomography Biomarkers Detected with EfficientNetV2B1 and ConvNeXt

**DOI:** 10.3390/diagnostics14010076

**Published:** 2023-12-28

**Authors:** Corina Iuliana Suciu, Anca Marginean, Vlad-Ioan Suciu, George Adrian Muntean, Simona Delia Nicoară

**Affiliations:** 1 Department of Ophthalmology, “Iuliu Haţieganu” University of Medicine and Pharmacy, 400012 Cluj-Napoca, Romania; scorinamail2019@gmail.com (C.I.S.); georgemuntean99@gmail.com (G.A.M.); simonanicoara1@gmail.com (S.D.N.); 2 Department of Computer Science, Technical University of Cluj-Napoca, 400114 Cluj-Napoca, Romania; 3 Department of Neuroscience, “Iuliu Haţieganu” University of Medicine and Pharmacy, 400012 Cluj-Napoca, Romania; vs_sib@yahoo.com; 4 Department of Ophthalmology, Emergency County Hospital, 400006 Cluj-Napoca, Romania

**Keywords:** OCT, state-of-the-art AI, diabetic macular edema screening, diabetes mellitus, ocular conditions, biomarkers

## Abstract

(1) Background: Diabetes mellitus (DM) is a growing challenge, both for patients and physicians, in order to control the impact on health and prevent complications. Millions of patients with diabetes require medical attention, which generates problems regarding the limited time for screening but also addressability difficulties for consultation and management. As a result, screening programs for vision-threatening complications due to DM have to be more efficient in the future in order to cope with such a great healthcare burden. Diabetic macular edema (DME) is a severe complication of DM that can be prevented if it is timely screened with the help of optical coherence tomography (OCT) devices. Newly developing state-of-the-art artificial intelligence (AI) algorithms can assist physicians in analyzing large datasets and flag potential risks. By using AI algorithms in order to process OCT images of large populations, the screening capacity and speed can be increased so that patients can be timely treated. This quick response gives the physicians a chance to intervene and prevent disability. (2) Methods: This study evaluated ConvNeXt and EfficientNet architectures in correctly identifying DME patterns on real-life OCT images for screening purposes. (3) Results: Firstly, we obtained models that differentiate between diabetic retinopathy (DR) and healthy scans with an accuracy of 0.98. Secondly, we obtained a model that can indicate the presence of edema, detachment of the subfoveolar neurosensory retina, and hyperreflective foci (HF) without using pixel level annotation. Lastly, we analyzed the extent to which the pretrained weights on natural images “understand” OCT scans. (4) Conclusions: Pretrained networks such as ConvNeXt or EfficientNet correctly identify features relevant to the differentiation between healthy retinas and DR, even though they were pretrained on natural images. Another important aspect of our research is that the differentiation between biomarkers and their localization can be obtained even without pixel-level annotation. The “three biomarkers model” is able to identify obvious subfoveal neurosensory detachments, retinal edema, and hyperreflective foci, as well as very small subfoveal detachments. In conclusion, our study points out the possible usefulness of AI-assisted diagnosis of DME for lowering healthcare costs, increasing the quality of life of patients with diabetes, and reducing the waiting time until an appropriate ophthalmological consultation and treatment can be performed.

## 1. Introduction

Diabetes mellitus (DM) has an important impact on the healthcare system, which in turn generates a need for screening measures and early preventive management to increase the patients’ quality of life. By 2030, because of the increasing prevalence of DM (366 million affected worldwide), there will be a higher burden on the healthcare system around the world and a negative effect on screening programs, impacting the timely evaluation of these patients. Diabetic retinopathy (DR) imposes an important vision-threatening risk in patients with diabetes <75 years old. The overall global prevalence of diabetic macular edema (DME), which can occur in any evolution stage of DR, is 6.8% according to Yau et al. According to the Diabetes Control and Complications Trial, 27% of the people with type 1 DM (T1DM) have DME in 9 years of disease evolution, while 13.9–25.4% of the people with type 2 DM (T2DM) will develop DME in their evolution [1,2,3,4].

Optical coherence tomography (OCT) provides cross-sectional scans of the retinal structure, and is used on a daily basis not only for research purposes but also in clinical practice to diagnose and follow-up ocular conditions before and during treatment [4].

In order to appropriately evaluate DME, frequent monitoring and follow-up is needed. To accomplish this, OCT has the advantage of being a non-invasive, non-irradiating imaging method able to quantify precisely the thickness of the retina in vivo and to display its structure at the microscopic level [5].

The OCT scanning technique uses infrared, non-ionizing rays that are safe and capable of imaging the morphology of the retina. Swanson and colleagues estimated a total of 30 million OCT image acquisitions performed worldwide each year [6].

In DME patients, the pathophysiology is complex, because oxidative stress not only affects the pericytes and endothelial cells but also influences the apoptotic process, determining neuronal loss of retinal ganglion cells (neurodegeneration). Chronic hyperglycemia activates inflammatory pathways at a cellular level, which in turn cause changes in the retinal microcirculation, generating abnormalities in the vessel structure (examples: microaneurysm, obstruction, thrombosis, intraretinal microvascular abnormalities–IRMA, irregular veins, and venous loops). These microvascular abnormalities lead to changes in blood flow, capillary hyperpermeability, edema, inflammation, chronic hypoxia in the retinal tissue, and the accumulation of metabolites, resulting in non-perfused areas and necrosis in the final stages [4,7,8,9,10,11,12,13].

Macular edema appears as a result of fluid leakage from the affected capillaries (micro-ischemia of the vessel walls) and thickening and disorganization of the retinal layers. Moreover, faced with this complex pathological aggression, vascular growth factors are produced in high quantities, determining vessel proliferation in order to compensate for the blood supply. However, these newly formed vessels are more fragile and prone to micro-hemorrhages. Their rupture and extravasation leads to the accumulation of blood between the retinal layers, deforming their normal structure (gliosis, retinal traction, and detachment) [4].

The hallmarks of DME are represented by [4,7,8]: A. Hyperreflective foci (HF) appear as hyperreflective (white) dots and represent lipoprotein deposits that precipitate and are localized at the limit between the normal and edematous retina in the internal layers. They are considered markers of retinal inflammation [4,7,8]. B. Edema and intraretinal cystic spaces (hyporeflective, dark) are also the consequence of blood–retina barrier (BRB) breakdown by increasing the permeability of capillaries. The cystic spaces are initially isolated, but due to the accumulation of liquid, they can extend, causing diffuse edema. Edema can alter the structure of the photoreceptor layer and cause severe visual impairment when affecting the macular region [4,7,8]. C. Detachment of the sub-foveolar neurosensory retina (prevalence 15–30%) is seen as a hyporeflective area located between the edematous retina (confluent cystic spaces) and the pigmentary epithelium. These changes affect the photoreceptor cells through the accumulation of liquid and cavitation [4,7,8].

Moreover, the epimacular membrane can appear in DME patients, although it is not a specific biomarker, representing a fibrous tissue (whitish on the surface of the retina) formed at the level of the internal limiting membrane that contracts and thickens and causing an increase in macular thickness through fluid accumulation that produces tension and folds [4,7,8].

Artificial intelligence (AI) algorithms are nowadays used more frequently for research purposes in time-consuming tasks. Human subjectivity in reviewing and diagnostic decision-making can be lowered by associating an AI-based evaluator in clinical practice. Additionally, novel biomarkers can be detected using an AI algorithm, being highly cost-effective [14]. According to Ting et al., the screening options for diabetic retinopathy (DR) include ophthalmoscopy, slit lamp biomicroscopy, retino-photography, teleretinal screening, or retinal video-recording [15].

The most important disadvantages of the mainstream screening programs worldwide are the lack of modern equipment, small number of ophthalmologists in relation to the population size, and high healthcare costs for screening large populations [16,17,18,19].

According to the projected statistics, in the future, DM will claim a high cost for patient care in most countries around the world due to the increase in prevalence (11.6% of the healthcare budget). DR screening programs are of paramount importance in preventing vision-threatening complications. To accomplish this, a large amount of resources, both human and costly equipment, is required [20].

Machine learning (ML) and especially deep learning (DL) algorithms have been tested for automatic detection of X-ray images and skin photographs for various conditions such as tuberculosis, melanoma, lymph node metastases, and others. Nowadays, modern AI platforms have multiple applications for a large variety of ophthalmologic conditions, such as refractive surgery, cataract, glaucoma, iris tumor, retinopathies (age-related macular degeneration, diabetic retinopathy, and retinopathy of prematurity), and others. Ophthalmologic diagnostics is based on image-capturing devices (example OCT, digital fundus photography, and visual field), and therefore are suitable for AI integration or assistance. Since the recent global pandemic, AI confers easy integration with telemedicine, giving future directions. Time-consuming tasks with analyses of thousands of images per second can be easily processed by AI-based systems for automated screening programs, providing high accuracy [15,20,21,22,23].

The aim of our research was to investigate the efficiency and usefulness of AI-assisted diagnosis of DME for screening purposes. Several state-of-the-art AI neural networks (EfficientNetV2 and ConvNeXt) were tested in order to compare the efficiency in selecting images with the disease and correctly identifying major disease patterns related to DME: intraretinal cystoid spaces, HF, and subfoveal neurosensory detachment (ND). The novelties of our work are multiple: (i) we studied what the selected networks “know” before any fine tuning of the differentiation between healthy and DR scans or of the similarity between left and right eye of one patient; (ii) we analyzed volumes of OCT images not only for disease detection but also for the concurrent detection of 3 biomarkers specific to the studied disease; (iii) we proposed a new metric for the evaluation of our biomarker models within a volume along the evaluations at the level of B-scans. The image analysis for all images used raw OCT images, without any external image adjustments or quality enhancers, having the intent to be as close as possible to the conditions of the daily clinical practice.

## 2. Materials and Methods

This is an observational, case–control study that used a dataset of 2980 OCT images, which were completely anonymized based on patients with diabetes mellitus along with macular edema, non-diabetic (age-related macular degeneration) AMD subjects, and healthy controls.

All subjects signed an informed consent form upon enrollment. For each OCT image, we assigned a code in order to maintain privacy and data protection. This study adheres to the Declaration of Helsinki and was approved by the Ethics Boards of the “Iuliu Haţieganu” University of Medicine and Pharmacy Cluj-Napoca.

The criteria for including patients in this study were: patients with diabetes over 18 years of age. Subjects who had associated glaucoma, advanced cataracts (severe opacity with only light perception), and optic neuropathies were not included in the study.

The Heidelberg Engineering Spectralis Spectral Domain-OCT device with the EyeTracking feature enabled (Heidelberg Engineering GmbH, Heidelberg, Germany) was used to examine all the enrolled subjects, and was performed with Heidelberg Spectralis^®^ OCT2 Module Infrared Reflectance (Spectralis Software version 6.10.5) with fast macular scan and thickness map rendering using 20 × 20° scan with 25 slices/retina at 200 µm and an automatic, real-time value of 9. The ETDRS map was rendered by the device software and used for clinical analysis.

**Figure 1:**
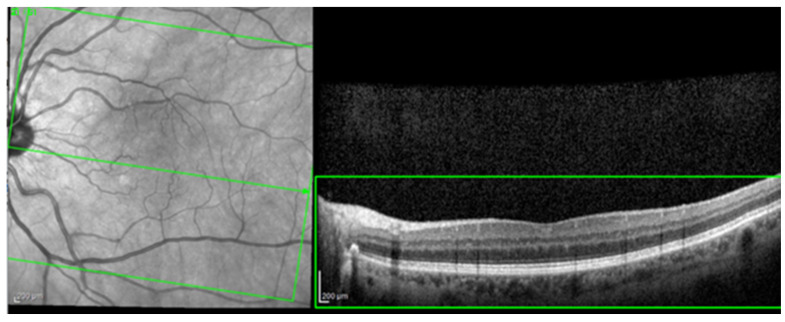
The selection of a region of interest (ROI) from an original optical coherence tomography (OCT) image (green box).

For the AI interface input, from the original OCT image acquisitions, a region of interest (ROI) was cut and standardized at a resolution of 256 × 256 pixels in order to have comparable input data for the software algorithm (see Figure 1).

Every selected ROI image was then again inspected by the same ophthalmologist for compliance. The OCT morphology analysis included the evaluation of cystoid spaces, HF, and subfoveal ND. In order to have “real-world” conditions, the training and testing of these algorithms was performed with OCT scans that included simple or complex (associated) patterns of disease (e.g., HF and cystoid spaces). Non-interpretable images with marked acquisition artifacts were analyzed separately.

**Figure 2:**
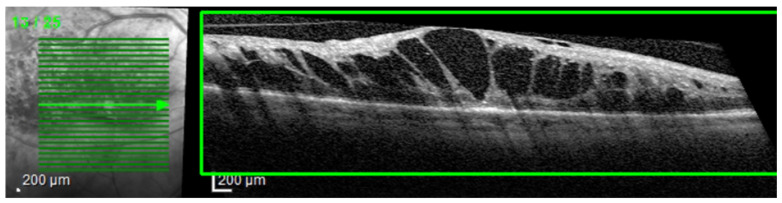
Image distortion. Observe the rhomboid aspect of the image slice (green box) giving the sense of a scan from an angle rather than a perpendicular scan to the retina’s base. This artifact is probably due to a sudden and rapid movement of the eye during scanning.

**Figure 3:**
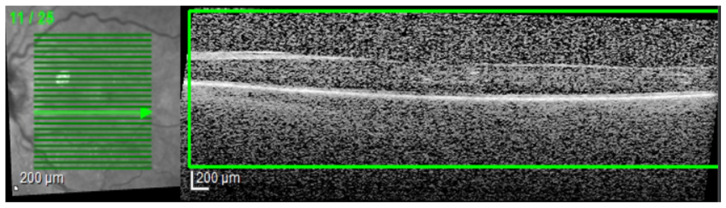
Image noise. Observe the snow-like appearance of the scan (green box). This artifact appears when the medium through which the infrared beams pass are not perfectly transparent (example: cataracts), making it very difficult to interpret the image.

The most common image artifacts observed were distortions and image noise. All images were included in the analysis, without having any external image adjustments or quality enhancers, in order to emulate “real world” everyday clinical practice conditions (see Figure 2 and Figure 3).

This study was performed in a series of steps. Firstly, the latent feature vectors (embeddings) from three pretrained architectures (EfficientNetV2B1, ConvNeXt tiny, and ConvNeXt base) were visualized with UMAP (Uniform Manifold Approximation and Projection for Dimension Reduction). Starting from the visualization-derived observations, the similarity between left and right eyes according to these embeddings was analyzed.

The next step was to train the selected architectures to correctly sort the input images into disease and healthy scans. The performance of the resulting models was described in terms of accuracy, precision, recall, F1-measure, and a new proposed metric.

**Figure 4:**
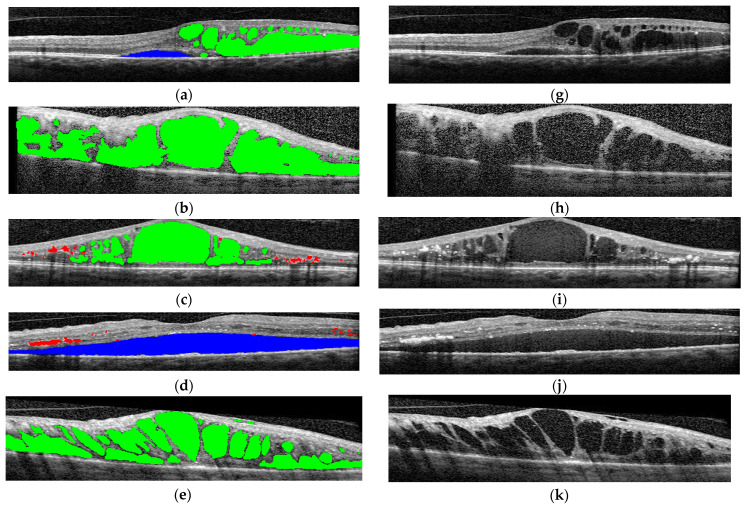

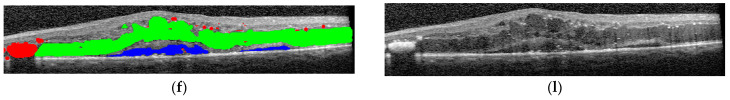
(**a**–**f**) Examples of pixel-level image annotations for training. The left column contains human annotated macular images, while the right column contains original OCT images. (**a**,**g**) edema and cystoid spaces; (**b**,**h**) edema and cystoid spaces; (**c**,**i**) cystoid spaces and hyperreflective foci (HF); (**d**,**j**) neurosensory detachment (ND) and HF; (**e**,**k**) cystoid spaces; (**f**,**l**) combined lesions. Green—edema and cystoid spaces; blue—ND; red—HF.

The final step was to use the selected architectures to identify the presence of different disease biomarkers (intraretinal cystoid spaces, HF, subfoveal ND, and neovascular AMD) in the images. In order to do this, all the images were annotated in terms of presence of the mentioned biomarkers. Apart from this, a smaller number of OCT images were manually annotated at the pixel level. All the annotations were performed at the OCT B-scan level, and not at the patient level (Figure 4). Nonetheless, the information about a patient being healthy or not is available.

Consequently, scans from the same patient can belong to different classes, including for example edema and detachment. The scan annotations included DME, ND, HF, others (choroidal neovascularization-CNV, bleeding, and epimacular membrane), artifacts, and healthy. More than one lesion can appear in the same image.

**Figure 5:**
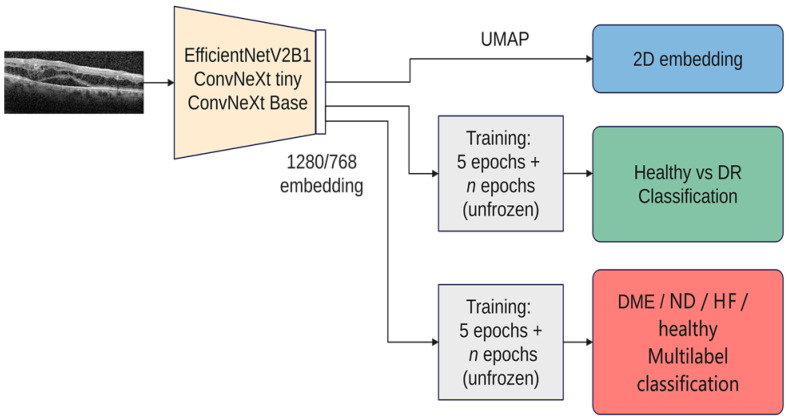
Overview of the experiments (DME—diabetic macular edema, HF—hyperreflective foci, ND—neurosensory detachment, UMAP—Uniform Manifold Approximation and Projection for Dimension Reduction).

Figure 5 gives an overview over our experiments. In all the experiments, we used three architectures: ConvNeXt tiny, ConvNeXt base, and EfficientNetV2B1. We chose these architectures due to their recent performance as well as the fact that ConvNeXt aims to benefit from both worlds, convolutional neural networks and transformer-based networks, while still remaining pure ConvNets base. Both ConvNeXt architectures have as input an image of the size 224 × 224, while EfficientNetV2B1 uses the size 240 × 240. The size of the feature vector for ConvNeXt tiny is 768, while for ConvNeXt base it is 1024, and for EfficientNetV2B1 it is 1280. The number of parameters for ConvNeXt tiny is ~27 M, while the base version has ~87 M; EfficientNetV2B1 has ~7 M parameters. EfficientNetV2B1 is much smaller than the ConvNeXt architectures. For the visualization with UMAP, all 3 networks used exclusively the ImageNet pretrained weights (Tensorflow 2.12). For training, the backbones are frozen the first 5 epochs, and then everything is trainable except the normalization layers. On top of the backbones, a dropout and a normalization layer are added, followed by the output layer. The tests were conducted on a dgx-1 system that featured 8 NVIDIA Tesla V100 GPUs with 32 GB of RAM each, requiring only a single GPU for the experiments. The inference using the smaller architectures (EfficientNetV2B1 and ConvNeXt tiny) was also run on a GeForce GTX 1080TI graphic card.

## 3. Results

### 3.1. Quantitative Analysis of the Dataset

The majority of the 52 patients have less than 200 images, except for two patients (C06 and M17). C06 had 240 scanned images in the DME-HF class, 141 in HF and 13 in healthy. Patient M17 had 164 scans in the DME-HF class and 99 in HF. Sixteen subjects had only healthy scans throughout. Fifteen patients had at least one healthy scan but also at least one scan with lesions. The majority of scans (95%) with macular edema also had HF.

**Figure 6:**
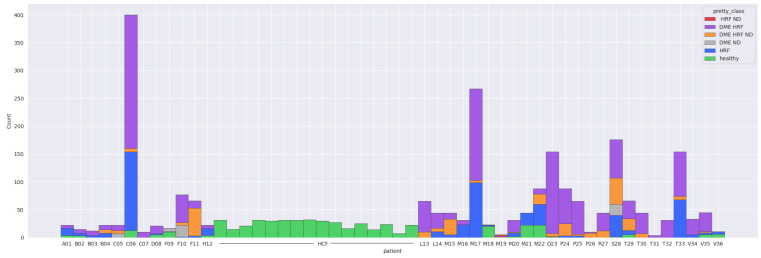
Distribution of scans.

Table 1 gives a detailed overview over the entire datasets. It can be observed that DME, HF, and ND appear in different combinations. Scans from CNV, ERM, and hemorrhages are all with DR combined with non-DR-specific lesions; therefore, for the current work, they are not considered in the rest of the experiments. Figure 6 shows the final data distribution of 2660 scans with healthy or combinations of DR-specific biomarkers.

Out of the 2660 scans, DME is present in 1615, ND in 330, and HF in 2096, while 510 scans are healthy. The average of biomarkers’ presence in DR scans is 1.87: 54 scans contain only HF, the rest of them have two or three biomarkers present. In terms of patients with at least one scan that presents one of the biomarkers (regardless of being the only biomarker): 34 patients have at least one scan with DME, 23 patients have at least one scan with ND, and 36 have at least one with HF. Thirty-one patients have at least one healthy scan, out of which sixteen are completely healthy (therefore all their scans are healthy), while fifteen present both healthy and DR scans.

### 3.2. Training/Test Split

Since the dataset contains scans from different patients, we first split the dataset into training and test, such that the training patients are disjointed to the test set of patients. We considered that a random split of the patients would not result in a representative training/test split due to large variation between the patients: (i) patients with only healthy scans, (ii) patients with all scans with DR, (iii) patients with DR and healthy scans, and (iv) different lesion combinations in DR scans. Therefore, we proposed a method where the patients are characterized by four binary features (F_patients_): three for biomarkers (DME, ND, and HF) and one for healthy. With this setting, it resulted a dataset made of 52 examples (the number of distinct patients), where an example is a patient, and there are four features: does the patient have at least one scan with DME (feature 0), does the patient have at least one scan with ND (feature 1), does the patient have at least one scan with HF (feature 2), and, finally, does a patient have at least one healthy scan (feature 3). We applied a training/test split on this dataset according to a multilabel classification problem and it resulted in P_train_ and P_test_. Table 2 presents the number of patients with different combinations of these features in the resulting training and test sets, respectively. The ratio between patients in training/test sets with different combinations of one or two F_patients_ features is between 0.13 and 0.21, except for patients with at least one ND and at least one healthy scan, where there is no such patient in the test set. Due to the large variability of patients, a perfect split with the same ratio for all the combinations is not possible, but we consider that the resulting split has an acceptable close distribution of patient types in the test set to the ones in the training set. The resulting patient sets P_train_ and P_test_ determine the scans that are going to be used in the rest of the experiments where learning is performed at the scan level.

### 3.3. 2D Visualization of Image Embeddings for the Entire Dataset

**Figure 7:**
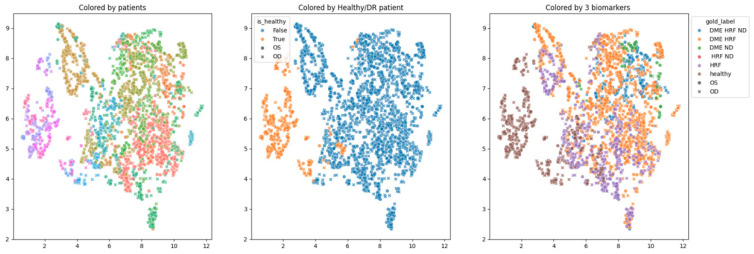
Two-dimensional UMAP visualization of ConvNeXt tiny (ImageNet pretrained weights) embeddings for the entire dataset.

**Figure 8:**
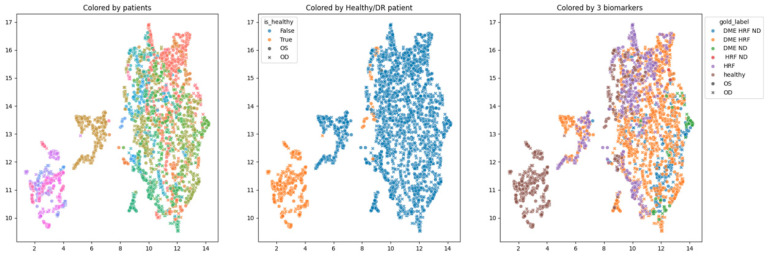
Two-dimensional UMAP visualization of ConvNeXt base (ImageNet pretrained) embeddings for the entire dataset.

**Figure 9:**
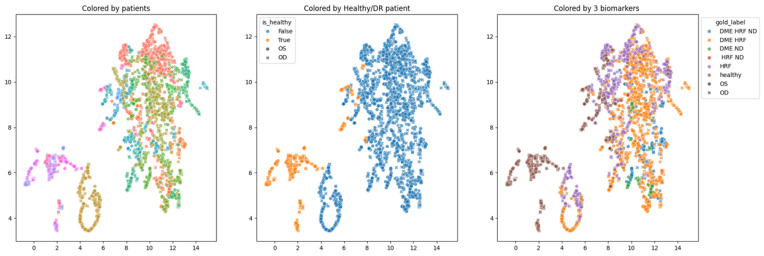
Two-dimensional UMAP visualization of EfficientNetV2B1 embeddings for the entire dataset.

In order to obtain an insight into how the pretrained networks represent the scans from our dataset, we applied UMAP dimensionality reduction to the embeddings obtained with the selected pretrained networks: ConvNeXt tiny, ConvNeXt base, and EfficientNetV2B1. This was conducted before any fine tuning, with the networks’ weights pre-trained on ImageNet. Figure 7, Figure 8 and Figure 9 show these visualizations. All three were obtained with 21 neighbors and cosine distance as parameters for UMAP. The questions behind these plots were: Are these embeddings capturing relevant information for differentiation between healthy and DR B-scans? What about the presence of HF, ND and DME?

Each of the three figures, Figure 7, Figure 8 and Figure 9, contains three images built with the same 2D UMAP embeddings, but with different color-coding schemes. In the leftmost image, the color is given by the patient, and so all the scans from one patient have the same color. In the middle image, the color is given by the type of patient: healthy or suffering from DR. The scans from healthy patients are orange, while all the scans (no matter whether they are healthy or not) from DR patients are blue. In the one on the right, the color is given by the type of scan, where the type can be healthy or combinations of HF, ND, and DME. The right eye (OD) is marked with round points, while the left eye (OS) is marked with an “x”.

Based on these visualizations, we draw several conclusions. First, the B-scans of the right and left eyes of the patients are quite close (the leftmost images). This could be due to the fact that the embeddings capture aspects that are particular to each patient but also that DR is a bilateral disease (the lesions in the right eye of one patient are closer to the lesions in the same patient’s left eye than to the lesions in other patients).

Secondly, the scans from healthy patients tend to be well separated from the scans from DR patients (the middle images). This conclusion will be supported also by the classification experiments. Lastly, the separation is not obvious anymore when we consider the three biomarkers (the rightmost images). 

We underline that in 2D UMAP, the relative position, not the exact position, is important. All these observations are analyzed with different experiments in the following subsections.

### 3.4. Similarity between Left and Right Eyes

In order to verify our observation that the embeddings obtained with the three pretrained networks of left and right eyes of one patient tend to be closer to each other than to the embeddings from other patients, we computed the cosine similarity between all left eye scans and all right eye scans for each patient (intrapatient similarity). For interpatient similarity for each patient, we computed the similarity between all of his/her scans and the scans from other patients. We underline that the scans from one patient are taken during several visits (different points in the disease evolution).

**Figure 10:**
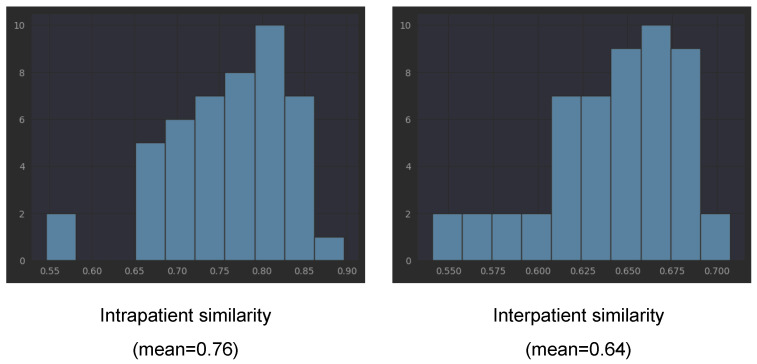
Similarity between EfficientNetV2B1 encodings (ImageNet pretrained).

Table 3 presents the means and standard deviations for cosine similarities computed with all three considered pretrained networks. The mean for EfficientNetV2B1 is 0.76 (with std = 0.073) for intrapatient similarity, while the interpatient similarity is smaller, 0.64 (with std = 0.03). Lower interpatient than intrapatient similarity is also present in the other two networks (see Table 3). This is not surprising if we consider that each patient has his/her own characteristics (present in both eyes, not exclusively disease-related). However, combined with the fact that the same embeddings capture the differentiation between healthy and not healthy, the fact that the intrapatient similarity is larger than the interpatient similarity could be a direct effect of DR bilateral involvement. The detailed histograms for EfficientNetB2B1 embeddings from Figure 10 shows that only in the case of two patients, the intrapatient similarity was smaller than 0.6. According to the three pretrained networks, patients V34, B03, and V35 have much smaller intrapatient similarity compared to the mean.

### 3.5. Metrics to Quantify the Quality of Predictions in Sequences (Volumes) of OCT Scans

**Figure 11:**
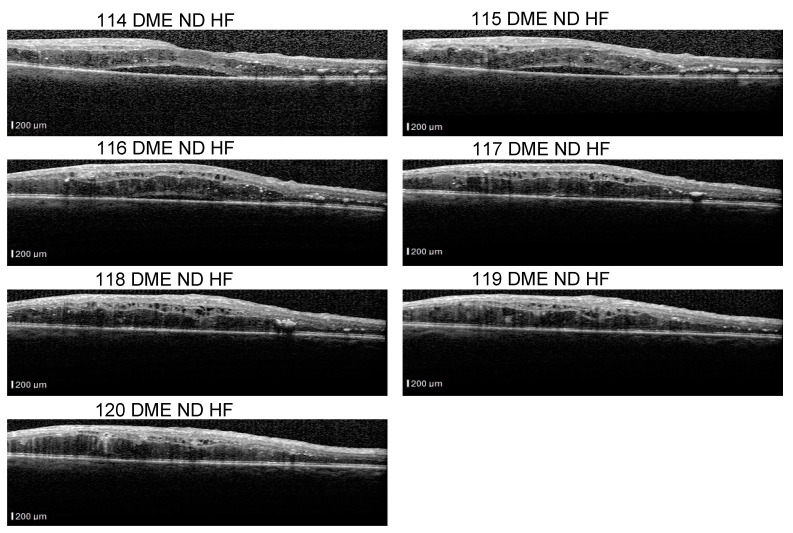
Scans from a sequence: ND is obvious in scans 114–117, and less obvious in scans 118–120.

Even though our dataset contains B-scans with scan-level annotations, these scans are part of volumes. The dataset does not contain complete volumes; therefore, we call them sequences. Figure 11 presents such scans that are part of a sequence: the detachment is obvious in 114–116 scans, but less obvious in scans 117–120. From the medical perspective, we consider that a model that identifies the detachment in at least one scan from all sequences that contain a detachment is better than a model that identifies all detachment scans in one volume but completely misses the detachment in another volume. At the same time, if the model is trained using a multilabel approach, it could be possible that, for example, in a sequence of DME combined with ND and HF, the model fails to identify correctly the complete combination in all the scans, but it detects DME and ND in one scan and DME and HF in another. 

The assumption of scan independence made by the common ML metrics computed at the scan level does not capture these aspects; therefore, we introduce new metrics that evaluate the model’s ability to signal the presence of biomarkers in a sequence (even though it might miss it in one scan from that sequence). Therefore, we introduce SeqAcc and SeqIoU, which are defined in the next paragraphs.

The fact that ND is very difficult to detect, for example, in scan 120 independent of the neighboring scans, and stresses also the difficulty of annotating such data. Hard examples (such as 118) could be extremely valuable for the models to learn to detect even the smallest signs of lesions.

(a) Label-based sequence metrics

Our biomarker model is trained in a multilabel manner: for each biomarker and healthy label, it predicts a probability. In all our experiments, we established 0.5 to be the threshold for considering the presence of the label. Classical accuracy for each label would count the ratio between all the correct predictions and all the scans. In order to capture the quality at the sequence level, we proposed the accuracy where the aggregation is first performed at the level of each sequence, and only after that, over all sequences. We call this sequence-based accuracy. For each label, for each sequence, SeqAcc^l^(seq) represents the ratio between the number of scans in that sequence with the correct prediction for label l and the total number of scans in the sequence. SeqAcc for the entire dataset is the mean over all sequences. We also compute a SeqAcc with exact matching for all four labels. The difference from the classical accuracy stands from the fact that an intermediate aggregation at the level of sequences is performed before the aggregation at the level of the entire set.

(1) $$SeqAcc^{l}\left(seq\right)=\frac{TP^{l}\left(seq\right)+TN^{l}\left(seq\right)}{TP^{l}\left(seq\right)+TN^{l}\left(seq\right)+FP^{l}\left(seq\right)+FN^{l}\left(seq\right)};SeqAcc^{l}=\frac{1}{seq\;no}\sum_{seq}SeqAcc^{l}\left(seq\right)$$

(b) IoU-based sequence metrics

SeqAcc is strongly affected by the imbalance between positives and negatives of each label. Even though for DR B-scans the co-occurrence of two biomarkers is almost two, since we have three biomarkers and also healthy scans, the negative examples for each label are significantly more than the positives (except for DME). In order to have an evaluation that is less affected by this imbalance and at the same time aiming to measure the model’s ability to detect the presence of biomarkers in the correct combinations, we consider a sequence-level metric based on intersection over union (IoU) or Jaccard index. Suppose c_s_ is the true set of labels for one scan s while ${\bar{c}}_{s}$ is the predicted set. IoU for one scan is the ratio between the number of correctly predicted labels and the size of the union between the predicted labels and the scan’s true labels. For example, if a scan has DME and ND and the model predicts DME and HF, IoU would be ⅓. SeqIoU(seq) of a sequence is the mean of IoU for scans in that sequence, while SeqIoU for the entire dataset is the mean IoU for all sequences.

(2) $$IoU\left(s\right)=\frac{\left|c_{s}\cap \bar{c_{s}}\right|}{\left|c_{s}\cup \bar{c_{s}}\right|};\;SeqIoU\left(seq\right)=\frac{1}{\left|seq\right|}\sum_{s\in seq}IoU\left(s\right);\;SeqIoU=\frac{1}{seq\;no}\sum_{seq}IoU\left(seq\right)$$

Since there are healthy patients and DR patients in the dataset, we also report SeqIoU for sequences of healthy patients and DR patients, respectively. For the former, the sequences contain only healthy scans, while for the latter, the three biomarkers and healthy scans can appear all in the same sequence. For consistency, we also report SeqIoU for Experiment 1 even though it follows a multiclass setup; consequently, IoU for each scan is either zero or one and SeqIoU is equivalent to SeqAcc.

### 3.6. Experiment 1—Multiclass Classification of Scans in Healthy vs. DR

The first type of experiments aimed for the classification of scans into two classes: healthy and DR. In all the following experiments, the same dataset split into training/test sets was used: the training set contains all the scans from patients in P_train_, while the test set contains all the scans from patients in P_test_. The number of DR scans in the test set is 236, while the healthy scans are fewer, 90. All the scans that include at least one biomarker were considered DR. In order to tackle the imbalancing effect, class weight was used during training with values of 0.8 for DR and 2 for healthy.

### 3.7. Experiment 2—Multilabel Classification of Scans with Three Biomarkers

Going beyond differentiation between healthy/DR, we aimed for finer grained classification of scans at the level of combinations of three biomarkers. This was formalized as multilabel classification. The possible labels are HF, ND, DME, and healthy. They are not mutually exclusive, and so the model will predict four independent values ranging from 0 to 1. Beyond the advantage of representing a combination of biomarkers, the multilabel approach also means that the model is not forced into choosing among the four possibilities. Therefore, in the case where none of the four labels are applicable, the model can predict zero for all four (for example, a scan with CNV but no DR).

We trained the same three backbones with an output layer of four neurons with sigmoid activation. The used loss is cross-entropy. The same training method is used as in Experiment 1: train for 5 epochs with the backbone frozen and learning rate 1e-2, and then train until early stopping with the unfrozen backbone and a learning rate of 3e-4 with the CosineDecay learning rate scheduler. The optimizer used for ConvNeXt was AdamW.

## 4. Discussion

A recent study of 303 eyes with DME, which evaluated OCT biomarkers through semi-supervised AI algorithms, concluded that the assisted diagnosis using AI is efficient and reproducible. Moreover, this method can be also used for determining the prognosis of DME patients [17]. The study [17] used pixel-level annotations, which are known to be time-consuming. Our work started from the assumption that since recent neural networks are known to perform well on the classification between healthy and non-healthy, they must be aware of biomarkers at least to some extent. Therefore, we aimed to learn to detect the presence of biomarkers from scan-level annotations. Furthermore, with the Grad-Cam technique, we also obtained the localization of these biomarkers.

### 4.1. Experiment 1—Classifying Scans as Healthy vs. DR

Table 4 presents the results for three pretrained networks, ConvNeXt tiny, ConvNeXt base, and EfficientNetV2B1, when trained to differentiate between healthy scans and DR scans. For each backbone, the results are presented (i) after the first 5 epochs during which the backbone is frozen (lines in the table with false in the last column) and (ii) at the end of the training given by early stopping (the training is continued from the 6th epoch with the backbone unfrozen and with a decreased learning rate) (lines with true in the last column). We include in the table precision and recall for both DR and healthy (H) classes. The number of wrong predictions is also given in columns 4 and 5, with differences between scans that come from healthy patients and scans from DR patients. Sensitivity or the true positive rate for DR is the recall for DR (marked in the table in red), while specificity, or the true negative rate, is the recall for class H (the blue cells in the table).

EfficientNetV2B1: After fine tuning: all the B-scans with DR are correctly identified. Nine healthy scans are predicted to be DR, one is from a healthy patient (HCF1), the remaining eight are from patients suffering from DR and consequently are part of sequences of scans that include at least one DR scan (patients B02 and D08; for clarity we refer to these eight scans as Set 1).

Before fine tuning (5 epochs with the backbone frozen): All the B-scans with DR are correctly identified, fourteen of the healthy scans are predicted to be DR: one from HCF1 right eye, five from HCF1 left eye, one from D08 right eye, four from right eye, and three from B02 left eye. The mean probability for the wrong predicted scans from the healthy patient is 0.7, while it is 0.99 for healthy scans predicted as DR from the DR patients. Therefore, even though the model misclassifies six healthy scans from healthy patients, it is less confident in its predictions than for the eight scans misclassified from DR patients. 

ConvNeXt tiny: After fine tuning, no scans from healthy patients are predicted as DR. The same eight healthy scans from Set 1 are predicted to be DR (exactly the same as with EfficientNetV2B1).

ConvNeXt base: After fine tuning, four DR scans are wrongly classified as healthy. The healthy images from Set 1 (all of them belong to DR patients), which consistently were classified by the other models as DR, were also misclassified as DR, except for one. 

In the case of all three networks, the sensitivity is one (with one exception), even after the first 5 epochs. Specificity obtained without fine tuning is consistently over 0.8 and it increases with fine tuning to values above 0.9, with ConvNeXt base achieving the best value of 0.92 together with a sensitivity of 0.98. Since the dataset is highly imbalanced, the accuracy gives no clear indication about the effective quality of the model. A more informative metric is SeqIoU for healthy individuals vs. DR patients: all three architectures improve SeqIoU for the healthy individuals after fine tuning since the number of misclassified healthy scans from healthy individuals is reduced to zero or one. The SeqIoU for DR patients is 0.95 for all (with or without fine tuning) except for ConvNeXt base that obtained 0.96 after fine tuning at the cost of misclassification as healthy of four DR scans with HF. 

The differences between the three learning experiments are not significant; they all support the known conclusion that the differentiation between healthy and DR OCT scans can be achieved with very good performance with DL. The novelty is that the three selected pretrained networks are capable of giving very good embeddings for this classification problem even without any fine tuning. We can observe that the performance after 5 epochs of training the networks with the backbone frozen are not significantly different from the result of fine tuning (i.e., the parameters in the backbone are changed during fine tuning when the backbone is unfrozen). This observation is in line with the observation derived from the UMAP 2D visualization of the embeddings (Figure 7, Figure 8 and Figure 9, the images in the middle).

### 4.2. Experiment 2: Three Biomarkers’ Classification

**Figure 12:**
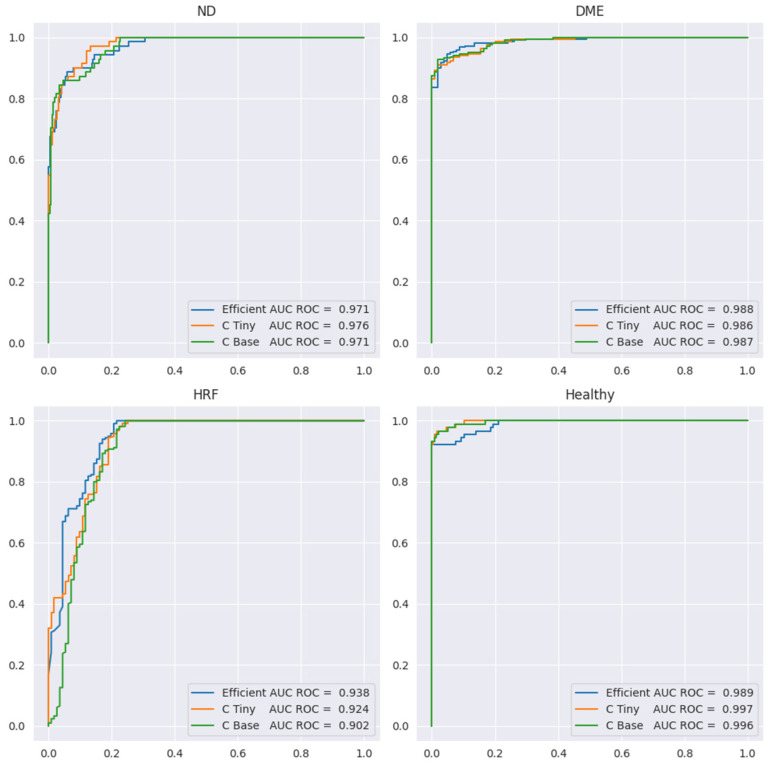
ROC curves for ND, DME, HF, and healthy in the multilabel setting.

The problem of the three biomarkers’ classification is more complex than the one of identifying healthy versus DR. For example, once a DME is identified, it is clear that the healthy class can be excluded even without understanding correctly the presence or absence of HF or ND. Therefore, it is expected to have smaller performance for the three biomarker models compared to healthy vs. DR. Table 5 presents the performance metrics, while Figure 12 shows the ROC curve for all four labels. Sensitivity or the true positive rate for each biomarker is the recall for the YES class for each biomarker (marked in the table in red). Specificity, or the true negative rate, is the recall for class NO for each biomarker (the blue cells in the table). We include here only the results for ConvNeXt base, since the results for the other two backbones are similar, but slightly lower.

Since the number of scans with ND is the smallest (71) and ND could be confused with DME, we preferred models that can correctly differentiate between these two aspects. In Table 5, we can observe that sensitivity for ND is 0.82 at a specificity of 0.97 when computed at the level of scans; however, SeqAcc is 0.94 for ND, meaning that when ND is present in a sequence, the model tends to correctly identify ND at least in one scan. For DME, the sensitivity is 0.92 at a specificity of 0.98. Even though F1 for DME is the largest among the three biomarkers, SeqAcc for DME is 0.91 (worse than SeqAcc for ND and healthy). This happens because there are some sequences of scans all with DME combined with HF where the model predicts only HF and misses DME. 

Unlike this, in the case of HF, the sensitivity is high (0.97) but the specificity is smaller (0.78). This happens due to two types of errors: (i) wrong differentiation between healthy and HF in scans that belong to sequences where only HF and healthy scans are present; (ii) there are two sequences of 10 scans where only DME and ND are present, but the model consistently predicts DME together with ND and HF. Despite the smaller value for the specificity of HF, SeqAcc for HF is not far from the SeqAcc of DME.

As expected, the healthy class is the best identified among the four. Even though the multilabel setting does not assume that healthy is exclusive with DME, ND, or HF, the model never predicts healthy together with one of the three biomarkers. Furthermore, the misclassification of the healthy class refers only to confusion between the healthy and HF classes.

In terms of SeqIoU, where for a scan we consider all four predictions, the value is 0.89, with 1 for healthy patients, meaning that the healthy sequences are identified completely correctly, and 0.85 for sequences from DR patients. Based on all these metrics, we can assess that the model can be used reliably to signal the presence of biomarkers in sequences of scans.

**Figure 13:**
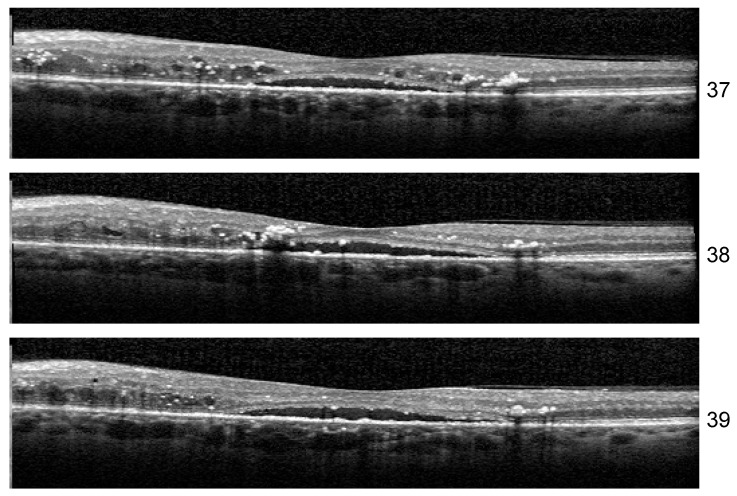
DME+HF+ND scans inside a sequence.

Stability: Unlike the results of Experiment 1, the misclassified examples change within different architectures or even the same architecture but different training. If Set 1 is consistently misclassified by all the runs of the Experiment 1 with one exception, in the case of three biomarkers, the errors in the identification of less obvious biomarkers varies along different runs. One such example is the HF scan from patient B02 left eye, slice 39, illustrated in Figure 13. The model for which we report the performance and Grad-Cam visualization identified it as healthy, but the same architecture, with a minor change in training, predicts that the image is not healthy but also that none of the biomarkers (DME, HF, or ND) is present.

Another curious behavior was observed for scan 38 from the sequence in Figure 13. There were runs from all the architectures that could correctly predict for all three scans the presence of all three biomarkers, but in more than one run, scan 38 was predicted only to have DME and HF while both neighboring scans were predicted as DME+HF+ND. The reason behind this is not yet clear, but we suspect that the presence of HF exactly near the detachment impairs the model’s ability to consider the presence of ND.

Even though the lack of complete stability indicated limitations in our study, which are mainly related to the size of the dataset, the large values for Seq IoU indicate that the “three biomarker” model is able to detect the presence of DME, ND, or HF in a volume, while potentially missing their presence in some B-scans from the volume.

### 4.3. Grad-Cam Visualization

Even though Grad-Cam or other methods for XAI on neural networks are known to have limitations, we consider them of high importance for the medical field and especially in our situation, in which we want the networks to learn to differentiate between different biomarkers based only on the scan level as opposed to pixel-level annotations. In our view, the most important limitation of heatmap oriented XAI methods is their intrinsic inability to express what is not present in an image, but it is important for the prediction.

**Figure 14:**
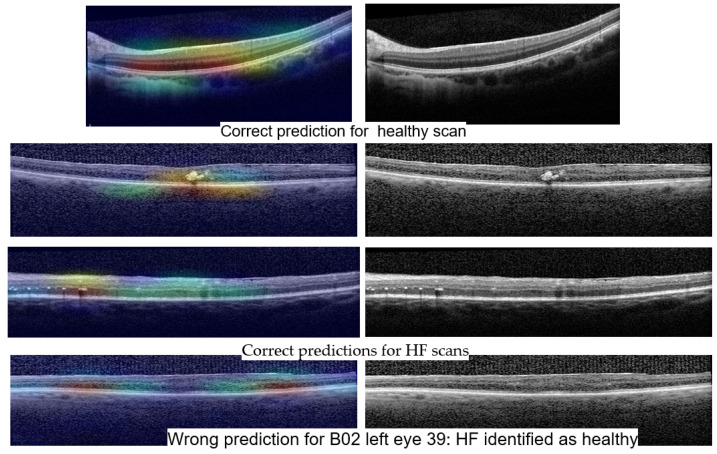
Grad-Cam visualizations for healthy and HF predictions with ConvNeXt base.

**Figure 15:**
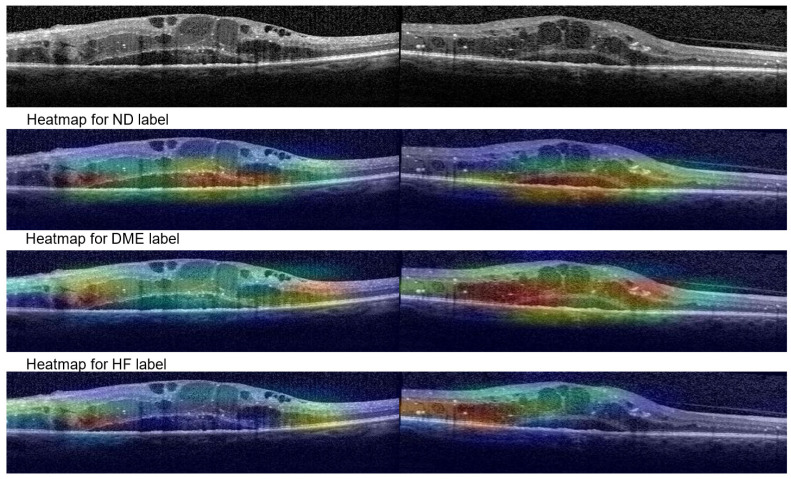
Grad-Cam visualizations for ND, DME and HF.

Figure 14 shows Grad-Cam heatmaps for three correct predictions: one healthy scan and two HF scans, and one HF+DME scan wrongly predicted as healthy. Figure 15 includes heatmaps for two scans that present all three biomarkers; therefore, we provide a heatmap for each label. The heatmap for the healthy scan shows that the entire area of the retina is important, while for the three biomarkers, restricted areas are more important. In all cases with correct predictions, the heatmaps indicate that the model is “looking” in the right area of the images and gives the correct interpretations for that area.

The wrongly classified HF+DME scan is consistent with the reported model’s performance. The model still has limitations and when the biomarker area is very small, the model can miss them.

When comparing the quality of the heatmaps generated with EfficientNetV2B1 with the ones generated with ConvNeXt base, the latter tend to be more precise. ConvNeXt base, with its ~87 M parameters, is larger than EfficientNetV2B1 that has ~7 M. In general, large number of parameters that need to be learned comes with the challenge of having a large training dataset. In our case, this challenge is alleviated by the fact that the pretrained weights are already very performant in identifying the presence of the disease (see Section 3.3 and Section 4.1). Beyond the architectural differences between EfficientNetV2B1 and ConvNeXt family, the superiority of ConvNeXt base comes also from the fact that the pretrained weights used for ConvNeXt base are the result of pretraining the model first on the ImageNet-21k dataset and then fine-tuning it on the ImageNet-1k dataset (https://www.tensorflow.org/api_docs/python/tf/keras/applications/convnext/ConvNeXtBase, accessed on 1 June 2023). On the other hand, training EfficientNetV2B1 has lower hardware requirements. One direction to be investigated is a more thorough comparison between the performance of the two architectures and different transformers for the task of biomarker identification and localization in a larger dataset.

### 4.4. Related Work

OCT images can be influenced by various factors, external to the image acquisition, which can damage the image quality. Wang et al. published in 2019 the results of their study in which they tested four different types of convolutional neural networks (VGG-16; Inception-V3; ResNet-18; and ResNet-50) for their effectiveness in assessing OCT quality and automatic detection. They concluded that ResNet-50 was the most accurate (96.25%). They classified the quality assessment into subjective and objective methods; the latter can automatically evaluate the retinal scan without any external intervention from the clinician [23].

According to Ting and colleagues, DL algorithms have wide implications in image processing, but despite this, they have only recently been introduced in healthcare. Machine learning, especially DL algorithms, have been tested for the automatic detection of X-ray images and skin photographs for various conditions, such as tuberculosis, melanoma, lymph node metastases, and others. In ophthalmology, DL has been researched for analyzing OCT images, retinal photography, and visual field images for diabetes-related complications, glaucoma, age-related macular degeneration, and retinopathy of prematurity. Because DL algorithms are advanced machine learning techniques and do not need manual intervention, these could change the way how ophthalmology will be perceived in the future. The effectiveness of different AI systems based on fundus photography for different retinal diseases has had sensitivities ranging from 84.2 to 100% and specificities ranging from 73.3 to 98.5%. In addition, the sensitivity ranges from 87 to 100% and specificity is 73.3–98.5% for DR, making it more suitable for screening [15]. Other authors developed similar AI models capable of predicting DR and DME with high accuracy on fundus images [16].

DR screening programs are of paramount importance in preventing vision-threatening complications, which require a large amount of resources, both humans and costly equipment. Through machine learning techniques, the screening procedures can be accelerated. According to statistics, DM will generate high costs in most countries (11.6% of the health budget). It has been shown that a high rate of false-positive results occurs when non-stereoscopic fundus photography is performed for the screening of DME (86% in Hong Kong; 79% in the United Kingdom). To increase the diagnostic accuracy, OCT is mandatory to be used in the screening of DME, mainly because DME is not easily screened on two-dimensional images. Furthermore, these authors stress the idea that DL with convolutional neural networks (CNN) using a hierarchy of artificial “neurons” and connections are able to interpret medical images with equivalent accuracy to human doctors [20].

Kermany et al. (2018) used OCT images for DL interpretation. Their study achieved a diagnostic accuracy of 98.2% with >90% sensitivity and specificity rates in classifying DME versus normal OCT images. Their model used over 11,000 OCT images for training and 250 OCT images for validation in classifying DME. They also performed a comparison between the AI model and six human experts in classifying four retinal diseases based only on OCT images, and concluded that the likelihood ratios between them was similar (CI 95%) [24].

A recent review on the subject of AI in the prediction of the DME response to anti-VEGF treatment showed that AI-based clinical tools can effectively enhance the therapeutic schedule for the patients and therefore improve management and their quality of life. Additionally, a digital health platform integration of AI could contribute to the prediction of the treatment response [25].

Perdomo et al. stress the fact that image analysis is more efficient in detecting microstructural changes when a feedback stage reveals to the clinician the region of interest on the OCT image. They used a CNN to scan the OCT volumes and achieved a high detection accuracy [26].

Venhuizen and colleagues developed an AI algorithm for detecting intraretinal cystoid spaces on OCT scans, achieving high performance in segmentation and quantification. It is important to specify that cropping and other image processing steps were used for image input and processing [27]. On the other hand, our research methodology did not use any image enhancements specially to test our algorithm under “real-world” conditions in which images can be distorted or non-ideal. We believe that for clinical conditions, only an AI algorithm that can prove its robustness and efficacy on most OCT scans has the potential to aid clinicians in the future, as patients differ from each other. Midena et al. stress the fact that OCT biomarkers are important for daily clinical practice, although they are subject to bias. It is of paramount importance to have a clinical tool for providing objective assessments. In this matter, AI has an important potential for automatic medical image analysis and contributes to clinical practice [28].

Li L. and colleagues consider that AI software is capable of recognizing with high efficiency minor fluid lesions in the retina based on OCT images, and are even better than human experts in some cases. Moreover, the disadvantages of a human image-by-image evaluation is time-consuming and subjective in the evaluation, making AI a more suitable candidate for screening purposes [29]. Roberts et al. developed an AI algorithm that can process and detect automatically and specifically fluid volumes on OCT images [30]. On the other hand, other authors, such as Okuwobi et al., developed software that is capable of specifically detecting only HF lesions in the retina [31,32]. Specific lesion detection is an important step forward in developing a complex AI model that can analyze and predict multiple lesions or even multiple conditions based on OCT images. The most important factor is to be reproducible and capable of using clinical, raw OCT images and to prove its robustness in the clinical setting. We strived to include raw OCT images and develop a multi-analysis model to be tested in real-life clinical settings.

## 5. Conclusions

Many AI architectures are used today for research purposes in medicine because of the processing speed of vast amounts of raw data. Clinical applications for AI may be disease screening, assisted diagnosis and decision-making. Promising steps are made every day in bringing AI-assisted diagnosis closer to being implemented as a clinical tool for physicians in order to make fast decisions in complex situations.

Our research is yet another argument that AI has potential in generating a useful tool for clinical applications, such as screening and diagnosing patients with diabetes mellitus for macular complications. Multiple lesion and condition recognition based on raw clinical data (non-preprocessed OCT images) with robust and reproducible AI algorithms is needed for clinical applications. Time-consuming tasks can be performed with the help of an AI algorithm in the future, although human, expert supervision is recommended in any case.

One important conclusion of our experiments is that pretrained networks such as ConvNeXt or EfficientNet capture features relevant to the differentiation between healthy and DR, even though they were pretrained on natural images. The fact that very good performance for the differentiation between DR and healthy scans can be obtained without changing the pretrained weights of these networks support this conclusion. Another conclusion is that the differentiation between biomarkers and their localization can be obtained even without pixel level annotation. Our three biomarker model is able to identify obvious ND, DME, or HF, but also very small ND. The main limitation of this study resides in the fact that the dataset is small, especially for the three biomarker model. Further work is needed in order to improve the performance of HF detection and to quantify the degree to which the localization of all HF or DME present in an image is correctly captured by the Grad-Cam visualization.

## Figures and Tables

**Table 1:** Number of B-scans for each patient.

Patient	HF	DME HF	DME HF ND	DME ND	HF	CNV + DR	ERM + DR	Hemorrhage DR	Artifact	Healthy	Not
	ND										Healthy
A01	0	0	5	0	0	0	13	0	0	4	18
B02	0	0	7	0	0	6	5	0	0	3	18
B03	0	7	8	0	0	0	4	3	0	0	22
B04	0	0	8	6	0	0	8	0	0	0	22
C05	0	0	9	6	7	0	0	0	0	0	22
C06	0	0	240	6	0	0	141	0	0	13	387
C07	0	0	10	0	0	0	0	0	0	0	10
D08	0	0	14	0	0	1	2	0	0	5	17
F09	0	5	0	0	6	0	1	0	0	10	12
F10	0	0	50	5	21	0	1	0	0	0	77
F11	0	0	13	50	0	0	3	0	0	0	66
H12	0	0	6	0	0	0	12	0	0	4	18
HCF1	0	0	0	0	0	0	0	0	0	31	0
HCF10	0	0	0	0	0	0	0	0	0	15	0
HCF11	0	0	0	0	0	0	0	0	0	21	0
HCF2	0	0	0	0	0	0	0	0	0	31	0
HCF3	0	0	0	0	0	0	0	0	0	30	0
HCF4	0	0	0	0	0	0	0	0	0	31	0
HCF6	0	0	0	0	0	0	0	0	0	31	0
HCF7	0	0	0	0	0	0	0	0	0	32	0
HCF8	0	0	0	0	0	0	0	0	0	30	0
HCF9	0	0	0	0	0	0	0	0	0	27	0
HCM1	0	0	0	0	0	0	0	0	0	16	0
HCM2	0	0	0	0	0	0	0	0	0	25	0
HCM3	0	0	0	0	0	0	0	0	0	14	0
HCM4	0	0	0	0	0	0	0	0	0	24	0
HCM5	0	0	0	0	0	0	0	0	0	7	0
HCM6	0	0	0	0	0	0	0	0	0	22	0
L13	0	0	55	10	0	0	0	0	0	0	65
L14	0	11	27	6	0	0	11	0	0	0	55
M15	0	0	11	28	0	0	5	0	0	0	44
M16	0	0	8	0	0	0	23	0	13	0	44
M17	0	0	164	4	0	0	99	0	0	0	267
M18	0	0	1	0	0	0	2	0	10	20	13
M19	2	115	2	1	0	0	0	14	0	0	134
M20	0	0	21	1	0	2	7	0	0	2	31
M21	0	0	0	0	0	0	22	0	0	22	22
M22	0	0	10	18	0	0	38	0	0	22	66
O23	0	0	147	5	0	0	1	0	0	1	153
P24	0	0	63	22	0	0	3	0	0	0	88
P25	0	0	59	3	0	0	3	0	0	0	65
P26	0	0	2	8	0	1	0	0	0	0	11
R27	0	0	32	12	0	0	0	0	0	0	44
S28	0	0	69	47	20	0	39	0	0	1	175
T29	0	44	32	21	0	0	8	0	0	5	105
T30	0	0	37	7	0	0	0	0	0	0	44
T31	0	0	4	0	0	0	0	0	0	0	4
T32	0	20	31	0	0	13	0	24	0	0	88
T33	0	0	80	6	0	0	68	0	0	0	154
V34	0	27	28	0	0	0	5	2	4	0	66
V35	0	0	34	2	0	0	4	0	0	5	40
V36	0	0	0	0	0	0	5	0	0	6	5
Total	2	229	1287	274	54	23	533	43	27	510	2472

**Table 2:** The number of patients in training/test datasets with different combinations of F_patient_ features [e.g., 0, 3 means patients that have at least one scan with DME (feature 0) and at least one healthy scan (feature 3)].

Combinations of *F_patient_*	0	0, 3	2, 3	0, 2	3	2	0, 1	1, 2	1	1, 3
Dataset										
# in P_train_	28	11	13	28	26	30	19	19	19	8
# in P_test_	6	2	2	6	5	4	4	4	4	0
test/train ratio	0.21	0.18	0.15	0.21	0.19	0.13	0.21	0.21	0.21	0

**Table 3:** Intra and inter cosine similarity between scan embeddings (mean and std).

	EfficientNetV2B1	ConvNeXt Tiny	ConvNeXt Base
intra patient	0.76 (0.07)	0.84 (0.04)	0.78 (0.09)
inter patient	0.64 (0.03)	0.79 (0.03)	0.70 (0.06)

**Table 4:** Performance for the classification into healthy and DR.

Precision	Recall	Seq_IoU Mean (Std)	Scans Wrongly Predicted as DR Healthy/DR Patient	Scans Wrongly Predicted as Healthy	Seq_IoU for DR Patients Healthy/DR Patient	Acc	Network	With Fine- Tuning
DR: 0.96 H: 1	DR: 1 H: 0.90	0.95 (0.02)	1/8	0	0.95/0.95 (0.01)/(0.02)	0.97	Efficient NetV2B1	True
DR: 0.94 H: 1	DR: 1 H: 0.84	0.93 (0.03)	6/8	0	0.88/0.95 (0.04)/(0.02)	0.96		False
DR: 0.97 H: 1	DR: 1 H: 0.91	0.96 (0.02)	0/8	0	1/0.95 (0)/(0.02)	0.98	ConvNeXt tiny	True
DR: 0.94 H: 1	DR: 1 H: 0.82	0.95 (0.02)	8/8	0	0.92/0.95 (0.01)/(0.02)	0.95		False
DR: 0.97 H: 0.95	DR: 0.98 H: 0.92	0.95 (0.02)	0/7	4 (2 HF + 2 DME& HF)	1/0.94 (0)/(0.03)	0.97	ConvNeXt base	True
DR: 0.95 H: 1	DR: 1 H: 0.87	0.95 (0.02)	4/8	0	0.96/0.95 (0.003)/(0.02)	0.96		False

**Table 5:** Performance metrics for ConvNext base (SeqAcc with exact matching for all labels = 0.80).

Label		Support	Precision	Recall	F1	Roc Auc	SeqAcc	Seq IoU	Seq IoU_H_	Seq IoU_DR_
ND	no	255	0.95	0.97	0.96	0.97	0.94	0.89	1 (0)	0.85 (0.03)
	yes	71	0.89	0.82	0.85					
DME	no	104	0.86	0.98	0.91	0.98	0.91			
	yes	222	0.99	0.92	0.96					
HF	no	111	0.94	0.78	0.85	0.90	0.90			
	yes	215	0.90	0.97	0.93					
Healthy	no	236	0.99	0.97	0.98	0.99	0.96			
	yes	90	0.94	0.97	0.95					

## Data Availability

The data presented in this study are available on request from the corresponding author. The data are not publicly available due to privacy.
